# Mini-Sternotomy for Aortic Valve Replacement with Concomitant Ablation of the Pulmonary Vein and Persistent Left Superior Vena Cava: A Case Report

**DOI:** 10.21470/1678-9741-2024-0053

**Published:** 2025-04-28

**Authors:** Kensuke Kobayashi, Yusuke Mizuno, Takayuki Kai

**Affiliations:** 1 Department of Cardiac Surgery, Daiyukai General Hospital, Ichinomiya, Aich, Japan; 2 Department of Surgery, Daiyukai General Hospital, Ichinomiya, Aich, Japan

**Keywords:** Superior Vena Cava, Persistent Left Superior Vena Cava, Sternotomy, Aortic Valve Stenosis, Atrial Appendage, Tomography

## Abstract

A 79-year-old man with severe aortic valve stenosis and atrial fibrillation was referred to our department for surgery. Computed tomography revealed persistent left superior vena cava. Lower mini-sternotomy was performed. The left atrial appendage was amputated before ablation of the persistent left superior vena cava. The jaw of the ablation device was passed behind the pulmonary veins using a tip-lighted articulating dissector. Finally, aortic valve replacement was completed. Aortic valve replacement with concomitant pulmonary vein and persistent left superior vena cava ablation via lower mini-sternotomy is a safe and less invasive alternative.

## INTRODUCTION

**Table:** 

Abbreviations, Acronyms & Symbols	
GP	= Ganglionic plexus
LAA	= Left atrial appendage
LED	= Light-emitting diode
PLSVC	= Persistent left superior vena cava
PV	= Pulmonary vein

Minimally invasive aortic valve surgery via mini-sternotomy or small right thoracotomy is commonly performed. However, concomitant pulmonary vein isolation with aortic valve replacement via a minimally invasive approach is challenging^[1^,^2]^. Moreover, to our knowledge, concomitant ablation of persistent left superior vena cava (PLSVC) with aortic valve replacement via mini-sternotomy has not yet been described. Herein, we report the first case of surgical aortic valve replacement combined with radiofrequency clamp ablation of the pulmonary veins and PLSVC with concomitant left atrial appendage amputation via lower mini-sternotomy.

## CASE PRESENTATION

**Fig. 1:**
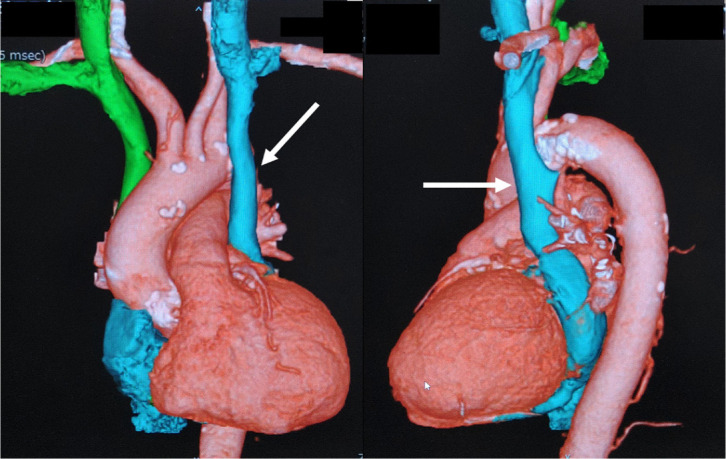
Three-dimensional computed tomography angiography. Arrows indicate persistent left superior vena cava.

A 79-year-old male with severe aortic valve stenosis was referred to our department for surgery. He had a history of paroxysmal atrial fibrillation and recurrent cerebral infarction. The patient had a height of 165.0 cm, weighed 65.0 kg, and his body surface area was 1.72 m^2^. The CHA2DS2-VASc score was 5. Transthoracic echocardiography showed an aortic valve area of 0.8 cm^2^, peak flow velocity of 5.3 m/s, and peak/mean pressure gradient of 110/66 mmHg. Computed tomography revealed a PLSVC draining into the coronary sinus and absence of the left brachiocephalic vein (Figure 1). The predicted perioperative mortality was 1.16% based on the European System for Cardiac Operative Risk Evaluation (or EuroSCORE) II and 2.43% based on the Society of Thoracic Surgeons Score (or STS SCORE). After anesthetic induction, transesophageal echocardiography showed no left-to-right shunt flow through the coronary sinus.

### Surgical Technique

The main surgical procedures are described below (Video 1).

**Video 1:**
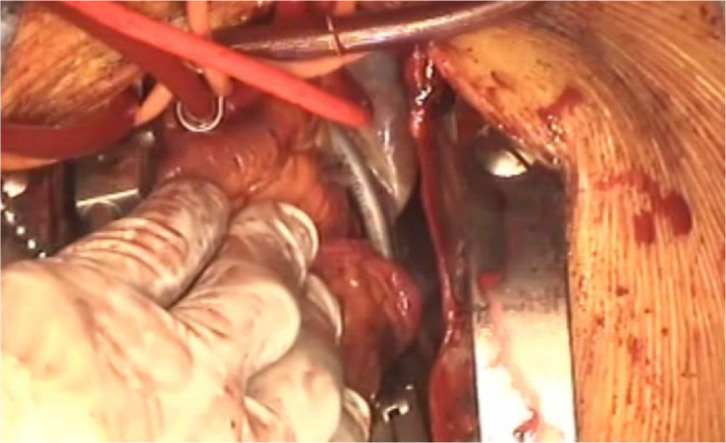
*Video of the main surgical procedures.*

*Link: https://youtu.be/5Z9uxFd5iBQ*

**Fig. 2:**
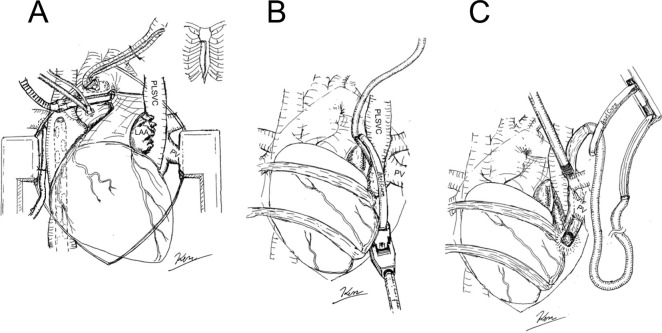
Schematic illustration of the procedure. Adequate exposure was achieved via lower mini-sternotomy, exiting through the right second intercostal space (A). The left atrial appendage (LAA) was amputated before persistent left superior vena cava (PLSVC) ablation (B). A tip-lighted articulating dissector was useful for preparing clamping procedures by scooping the guiding tape attached to the clamping jaw (C). PV=pulmonary vein.

**Fig. 3:**
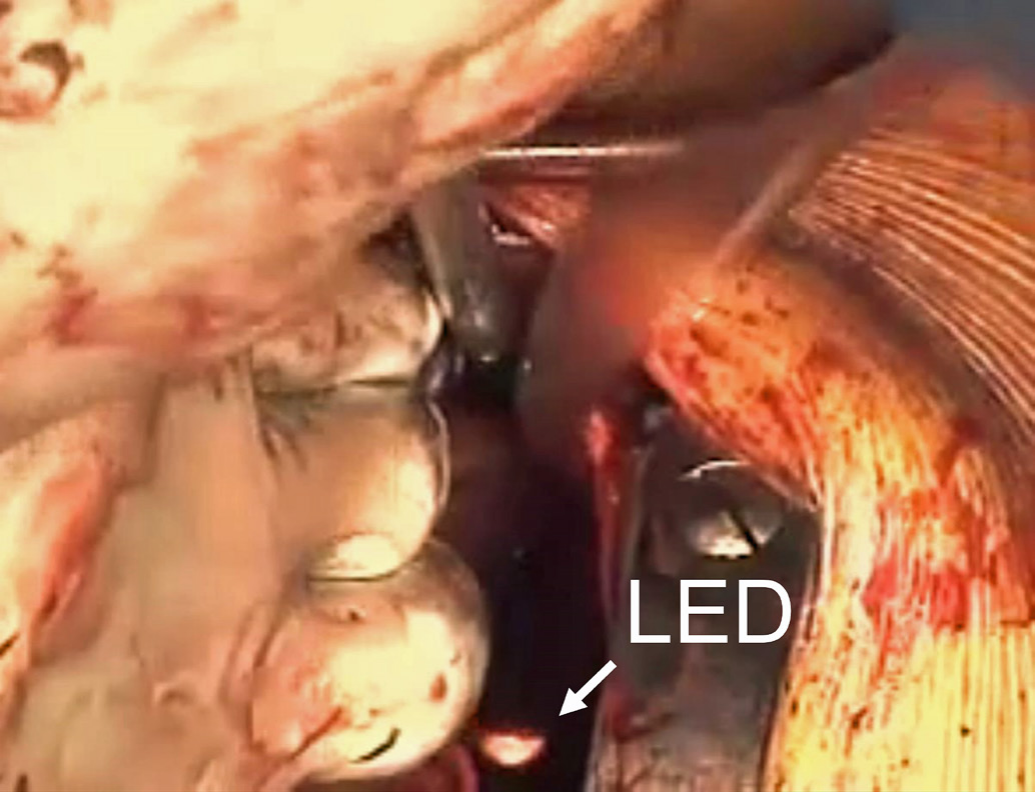
Left pulmonary vein isolation. The clamping jaw was passed behind the left pulmonary veins using an articulating dissector with light-emitting diode (LED) tip.

Lower mini-sternotomy was performed, exiting through the right second intercostal space. A cardiopulmonary bypass was established with ascending aortic cannulation and transfemoral right atrial drainage (Figure 2A). Ablation lines were made using the Isolator Synergy Access (AtriCure, Inc., Mason, Ohio, United States of America), which has an articulating flexible clamping head. Initially, on the beating heart, clamp ablation of the right pulmonary veins was performed. Then, a left ventricular vent was placed via the right superior pulmonary vein. Cardioplegic solution was infused, initially, from the aortic root cannula, followed by selective coronary perfusion after aortotomy. After cardioplegic arrest and decompression, the left atrial appendage was amputated using the Powered Echelon Flex with the GST60G stapler (Ethicon, Inc., Cincinnati, Ohio, United States of America), then the PLSVC was ablated (Figure 2B). The jaw of the clamping head was passed behind the left pulmonary veins, but the restricted surgical exposure made handling any type of forceps difficult. The Wolf Lumitip Dissector (AtriCure, Inc., Mason, Ohio, United States of America), a tip-lighted articulating dissecting device, was useful for preparing clamping procedures by scooping the guiding tape attached to the clamping jaw (Figures 2C and 3). Finally, aortic valve replacement with a bioprosthetic valve was completed in a standard fashion. The postoperative course was uneventful, and the patient was discharged on the 20^th^ postoperative day with sinus rhythm. Three months after the surgery, echocardiography showed a left ventricular ejection fraction of 61% and normal prosthetic valve function. The patient recovered well enough to go cycling.

This case study was conducted based on the Declaration of Helsinki and with the approval of our local Institutional Review Board (approval number: 2023-029). The authors obtained written publication consent for the case details and images.

## DISCUSSION

The embryological left superior vena cava regresses to the ligament of Marshall. Normally, the ligament of Marshall contains sympathetic ganglionic plexus (GP), which is one of the five major left atrial autonomic GPs^[3]^. The ligament of Marshall is commonly known as a major arrhythmogenic source of atrial fibrillation and is a potential target for ablation therapy. In PLSVC, the embryological left superior vena cava fails to regress and becomes a major source of venous return to the right atrium. The incidence of PLSVC in the normal population is 0.21%^[4]^. The incidence in congenital heart disease patients was reported as 5.9%, and a high incidence level up to 23.7% to 24.6% was observed especially in patients with coarctation of the aorta and double outlet right ventricle, respectively^[4]^. The GPs surrounding the PLSVC are similar to those surrounding the ligament of Marshall. The left superior vena cava-left atrial GP of porcine extends from the dorsal aspect of the left atrium to the medial origin of the left superior vena cava^[5]^. The PLSVC is electrically connected to both the left and right atria with potential arrhythmogenicity^[6]^. Simultaneous catheter ablation of the PLSVC and pulmonary veins is feasible^[6^-^8]^; however, an ablation strategy has not been established. When deciding on the indication for PLSVC ablation, there is controversy over whether to evaluate PLSVC as an arrhythmogenic source, *i.e.*, a trigger or driver for atrial fibrillation. There is a multicenter retrospective study on the efficacy of PLSVC ablation for long-term freedom from atrial fibrillation^[9]^. Chen et al.^[7]^ suggested performing PLSVC ablation empirically. Catheter ablation is commonly considered a safer minimally invasive procedure than surgical ablation. However, Wissner et al.^[8]^ reported a high incidence of severe complications related to catheter PLSVC ablation, such as cardiac tamponade and left phrenic nerve damage. Thoracoscopic surgical ablation for pulmonary veins and PLSVC with concomitant left atrial appendage amputation is another alternative minimally invasive intervention for atrial fibrillation^[10]^. The procedure can be safely performed even if catheter ablation is difficult because of the anomalous connection of the inferior vena cava in visceral situs inversus^[10]^. However, it is impossible to perform concomitant aortic valve replacement using total endoscopic surgery.

In an aging population, there is an increasing incidence of aortic valve stenosis with atrial fibrillation. In these cases, minimally invasive aortic valve replacement is performed; however, concomitant pulmonary vein ablation via minimally invasive approaches have rarely been reported^[1^,^2]^. Moreover, concomitant ablation of PLSVC via minimally invasive approaches have not been described. To our knowledge, this is the first reported case of aortic valve replacement combined with radiofrequency clamp ablation of the pulmonary veins and PLSVC via mini-sternotomy. Contrary to previous cases performed via upper mini-sternotomy^[2]^, we performed the procedure via lower mini-sternotomy, because the sufficient handling space was necessary for the clamp ablation procedures. A circular radiofrequency probe, such as the COBRA Fusion Ablation System (AtriCure, Inc., Mason, Ohio, United States of America), can enable a smaller approach^[2]^; however, it is unclear whether these devices can be used to create effective ablation lines for PLSVC. In aortic valve surgery via mini-sternotomy, upper mini-sternotomy is commonly preferred because it achieves excellent periaortic exposure. However, in this case, the surgical view of the aortic root was adequate even in lower mini-sternotomy. The safety and usefulness of this procedure needs to be evaluated over time.

## CONCLUSION

We have demonstrated the feasibility, safety, and curative ability of minimally invasive aortic valve replacement combined with radiofrequency clamp ablation of the pulmonary veins and PLSVC via lower mini-sternotomy.
